# Valved Femoral Vein Homografts as Right Ventricle to Pulmonary Artery Conduit for Repair of Truncus Arteriosus

**DOI:** 10.1016/j.atssr.2025.07.022

**Published:** 2025-08-20

**Authors:** Arif Selcuk, Justus G. Reitz, Mahmut Ozturk, Aybala Tongut, Manan Desai, Rittal Mehta, Irem Nida Turanalp, Sena Tuncer, Yves d’Udekem, Can Yerebakan

**Affiliations:** 1 Division of Cardiac Surgery, Children's National Hospital, The George Washington University School of Medicine and Health Sciences, Washington, DC; 2 Department of Cardiac Surgery, Nationwide Children’s Hospital, The Ohio State University College of Medicine, Columbus, Ohio

## Abstract

**Background:**

To assess outcomes of early surgical repair of truncus arteriosus by analyzing outcomes with the use of valved femoral vein homografts as right ventricle to pulmonary artery conduit.

**Methods:**

Between 2002 and 2023, 51 patients who underwent primary surgical repair for truncus arteriosus were reviewed retrospectively.

**Results:**

Median (interquartile range) age and weight were 7 (5.7-17.5) days and 3 (2.6-3.7) kg. Median follow-up was 7 (3-17) years. Survival was 86% and 81% at 1 and 15 years, respectively. Birth weight below 2 kg, conduit size of 10 mm or smaller, and the need for extracorporeal membrane oxygenation were associated with higher mortality. Valved femoral vein homografts were utilized in 29 (57%, 29 of 51) patients, pulmonary artery homografts in 11 (22%, 11 of 51), aortic homografts in 7 (14%, 7 of 51), valveless tube grafts in 3 (6%, 3 of 51), and a bioconduit in 1 (2%, 1 of 51). Thirty (59%, 30 of 51) patients underwent conduit replacement within a median of 2.7 (1.7-4.8) years. No difference was observed among conduit types based on the time from truncus arteriosus repair to conduit reintervention or replacement (*P* = .24). Freedom from conduit catheter-based reintervention or replacement was 75%, 59%, and 37% at 1, 2, and 3 years, respectively. Median conduit z-score was smaller in patients with conduit reintervention or replacement within 2 years compared with those after 2 years (2.78 vs 3.8, *P* = .04).

**Conclusions:**

Valved femoral vein homografts are durable as right ventricle to pulmonary artery conduits in early primary repair of truncus arteriosus. The choice of appropriately sized conduit reduces the rate of reintervention.

## Visual Abstract

**Figure:**
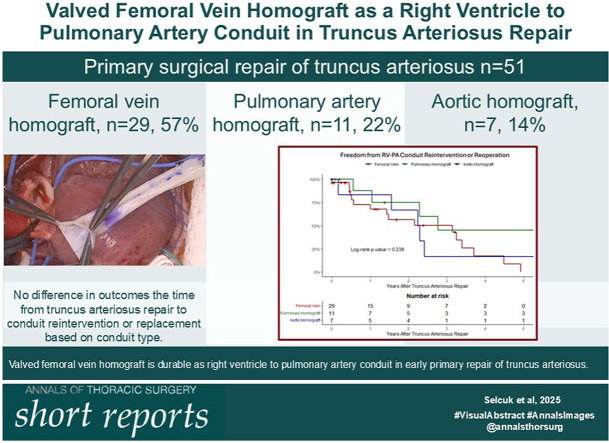

**In Short**

▪ Valved femoral vein homograft conduits are as effective as pulmonary artery and aortic homografts for reconstructing the right ventricular outflow tract in truncus arteriosus repair.
▪ They could serve as a feasible alternative to other right ventricle to pulmonary artery conduits in this setting.

Primary neonatal repair is the most widely accepted treatment for truncus arteriosus (TA), with low occurrence of persistent pulmonary hypertension and mortality, despite the eventual need for the right ventricle to pulmonary artery (RV-PA) conduit replacement.^1^ The RV-PA conduit remains a major cause of long-term morbidity,^2^ prompting ongoing efforts to optimize its type and size to delay reoperation. In this study, we sought to identify midterm outcomes of TA repair using valved femoral vein homografts as RV-PA conduit.

## Patients and Methods

### Patients and Study Design

The data of all consecutive patients who underwent repair of TA from 2003 to 2022 at Children’s National Hospital in Washington, DC, was retrospectively reviewed. The patients with hemitruncus (n = 5) or tetralogy of Fallot-pulmonary atresia-major aortopulmonary collateral artery (n = 8), or patients who underwent reoperation in our hospital after initial surgery in another hospital (n = 21) were excluded. The patients were categorized based on the type of RV-PA conduit used as follows: femoral vein homograft (LifeNet Health or CryoLife Inc), pulmonary artery homograft (CryoLife Inc), and aortic homograft (CryoLife Inc). The definitions and the clinical assessment processes have been summarized in the Supplemental Material for clarity. All available data were obtained retrospectively from institutional patient records. The Children’s National Hospital institutional review board approved the study, and waiver of informed consent was obtained (approval number and date: Pro00015566; July 1, 2021).

### Surgical Technique

The repair of TA was performed as previously described.^3^ RV-PA continuity was achieved using a femoral vein valve conduit, aortic or pulmonary homograft, or occasionally a valveless graft. Femoral vein homografts have been used since 2009, while most aortic and pulmonary homografts were used before 2009. For femoral vein homografts, it is important to check valve competency with saline before implantation, as the graft contains multiple valves, allowing for selection of one that is fully competent. To preserve valve configuration, the chosen valve should be positioned closer to the distal anastomosis. The conduit length should be carefully adjusted to prevent kinking. A line should be marked on the lateral or anterior surface to prevent twisting. TA repair using valved femoral vein homograft in RV-PA position is demonstrated in Supplemental Video 1.

### Statistical Analysis

Categorical variables are presented as number and percentage, and continuous variables are presented as median and interquartile range (IQR). Categorical variables were assessed using the χ^2^ test or Fisher’s Exact test, and continuous variables were evaluated using the *t* test or Mann-Whitney test as appropriate. Univariate analysis was performed to analyze demographic, perioperative, and follow-up data for mortality and reintervention on RV-PA conduit. Variables affecting reoperation or percutaneous reintervention on RV-PA conduit and mortality were tested using Cox regression analysis and comparison between conduit types was performed via log-rank testing. All assumptions including proportional hazards were assessed and necessary transformations were applied. Survival and freedom from reintervention on RV-PA conduit and conduit replacement were obtained by means of the Kaplan-Meier method with numbers at risk displayed and 95% CI obtained. Data were analyzed with the RStudio software version 4.3.1 (RStudio Team, 2020). Statistical significance was defined as *P* < .05.

## Results

Over a 20-year period, 51 consecutive patients underwent complete repair of TA. Femoral vein homografts were implanted in 29 (57%) patients, pulmonary artery homografts in 11 (22%), aortic homografts in 7 (14%), and other types of grafts in a total of 4 patients (8%), including 1 bioconduit and 3 valveless tube grafts. Demographic and perioperative data are presented in Supplemental Table 1.

### Survival

**Figure 1:**
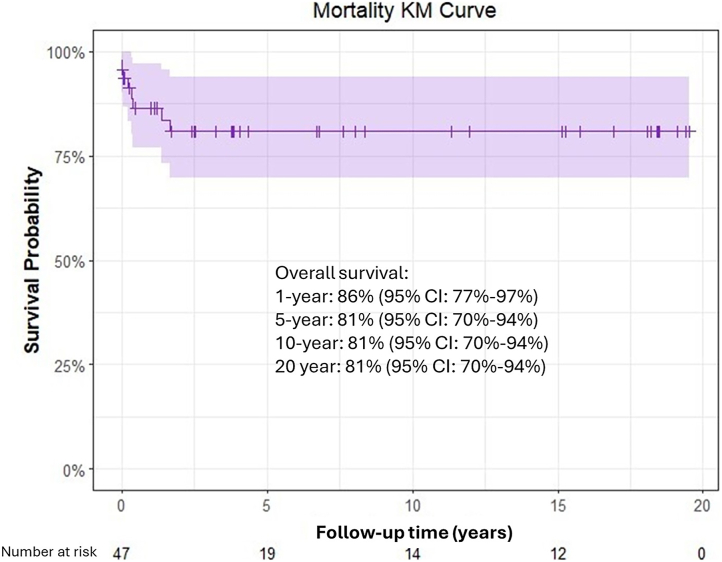
Kaplan-Meier (KM) survival curve after surgical repair of truncus arteriosus.

There were 45 (88%, 45 of 51) early survivors to discharge. Survival was 86% (95% CI, 77%-97%) at 1 year and 81% (95% CI, 70%-94%) at 5, 10, and 15 years (Figure 1). Survival rates for patients implanted with a femoral vein homograft as an RV-PA conduit were 90% at 6 months, 86% at 1 year, and 83% at 2 years. Among the 6 in-hospital deaths, 4 were associated with a cerebrovascular accident after extracorporeal cardiopulmonary resuscitation, 1 resulted from respiratory infection and persistent respiratory failure, and 1 was attributable to the development of low cardiac output. Bivariable analysis revealed the risk factors associated with mortality were the need for extracorporeal membrane oxygenation (hazard ratio, 17.8; 95%CI: 5-69; *P* < .01) and birth weight less than 2 kg (hazard ratio, 5.1; 95%CI, 1-25; *P* = .04) (Table 1).

**Table 1:** Bivariable Analysis of Factors Associated With Mortality

Variables	Hazard Ratio	95% CI	*P* Value
Antenatal diagnosis	1.96	0.49-7.86	.34
Prematurity	3.11	0.8-12.5	.11
<2 kg body weight at birth	5.05	1.03-24.9	**.04**
LV systolic dysfunction before TA repair	3.01	0.36-25.2	.31
<2.5 kg or less body weight at TA repair	2.95	0.73-11.9	.13
Concomitant aortic arch repair	2.38	0.59-9.52	.22
Need for extracorporeal membrane oxygenation	17.8	4.61-69.1	**<.001**
RV-PA conduit reintervention within 6 mo	1.22	0.25-5.89	.80
Preoperative significant truncal valve insufficiency	2.61	0.65-10.5	.18
Conduit type (femoral vein homograft vs aortic homografts)	1.03	0.28-3.85	.96

Bold highlights significant results.

LV, left ventricle; RV-PA, right ventricle-pulmonary artery; TA, truncus arteriosus.

### Reintervention on RV-PA Conduit

Catheter-based reintervention on RV-PA conduit prior to conduit replacement was performed in 14 (27%, 14 of 51) patients after a median of 9.4 (IQR, 5.8-26) months. These reinterventions included conduit balloon angioplasty in 7 patients and stent angioplasty in another 7 patients. There was no statistically significant difference among the types of RV-PA conduits regarding the proportion of catheter-based reinterventions or the time from the index surgery to the first catheter-based reintervention (*P* = .6, *P* = .77, respectively).

**Figure 2:**
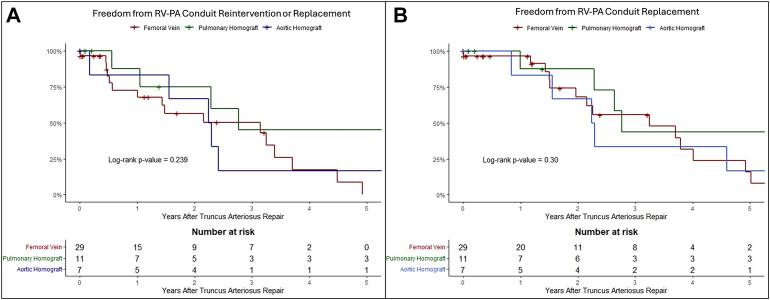
(A) Kaplan-Meier curves illustrating freedom from right ventricle-pulmonary artery (RV-PA) conduit reintervention or replacement by conduit type. (B) Kaplan-Meier curves illustrating freedom from RV-PA conduit replacement by RV-PA conduit type.

Thirty (59%, 30 of 51) patients underwent conduit replacement after a median of 2.5 (IQR, 1.5-4.6) years. Among the remaining 21 patients who did not undergo conduit replacement, 8 died within 9 months after TA repair, 5 were lost to follow-up, and 8 patients who underwent TA repair recently are being followed-up with their primary RV-PA conduit. Freedom from RV-PA conduit replacement and catheter-based reintervention was 75% (95% CI, 62%-91%), 17% (95% CI, 7%-40%), and 4% (95% CI, 1%-28%) at 1, 5, and 10 years, respectively (Figure 2A). Postoperative data are detailed in Supplemental Table 2.

A second conduit replacement was required in 9 patients at a median of 7.2 (IQR, 5.1-9.9) years after the first conduit replacement, and a third conduit replacement was performed in 3 patients at a median of 10 (IQR, 7.2-12.8) years after the second replacement. No femoral vein homograft was used during conduit replacement. There was no operative mortality at reoperations for conduit replacement.

Fifteen of the 29 (52%) patients with femoral vein conduit underwent conduit replacement within 2.3 (IQR, 1.5-3.9) years. Conduit replacement was seen in 7 (64%, 7 of 11) patients with pulmonary artery homograft within 2.8 (IQR, 2.5-7.8) years, and in 6 (86%, 6 of 7) patients with aortic homograft within 2.3 (IQR, 1.7-4) years. There was no difference between conduit types and time to conduit replacement (*P* = .3) (Figure 2B). Median follow-up time was longer in patients with aortic or pulmonary artery homografts compared with patients with femoral vein homografts (18 [IQR, 16-20] years, 16 [IQR, 1-19] years, and 3.8 [IQR, 1.3-7.2] years, respectively, *P* = .01). There was no statistically significant difference between the type of conduit and the time from TA repair to the first conduit reintervention or replacement (*P* = .24) (Figure 2A).

The analysis of time from TA repair to the first RV-PA conduit reintervention or replacement showed no statistically significant difference based on conduit size (greater or less than 55 mm/m^2^ and above or below +3 z-score), or conduit type (Table 2). Detailed follow-up data based on the type of RV-PA conduit can be found in Supplemental Table 2.

**Table 2:** Median Time to First RV-PA Conduit Reintervention (Catheter-Based or Surgical) Based on Conduit Size and Type

RV-PA Conduit Size	Median (IQR) Time to First Conduit Reintervention or Replacement, y	*P* Value for Size of Conduit	Type of RV-PA Conduit	Number of Patients	Median (IQR) Time to the First Conduit Reintervention or Replacement, y	*P* Value for Conduit Type
In all sizes	2.2 (0.6-3.4)		Femoral vein	29	1.5 (0.5-3.3)	.06
			Pulmonary artery	11	2.8 (1.7-7.8)	
			Aortic	7	2.3 (1.7-2.4)	
<55 mm/m^2^	1.3 (0.5-2.3)	.05	Femoral vein	15	0.6 (0.5-1.2)	.16
			Pulmonary artery	7	2.3 (1.7-4.1)	
			Aortic	4	1.9 (1.2-2.3)	
>55 mm/m^2^	3.3 (1.8-4.7)		Femoral vein	14	3.3 (1.4-3.7)	.3
			Pulmonary artery	4	6.3 (2.2-11.7)	
			Aortic	3	4.5 (3.4-5.6)	
<3 z-score	1.5 (0.9-2.3)	.14	Femoral vein	14	1.0 (0.5-1.5)	.3
			Pulmonary artery	6	2.3 (1.7-4.1)	
			Aortic	5	1.9 (1.2-2.3)	
>3 z-score	2.8 (0.6-4.5)		Femoral vein	15	2.2 (0.5-3.6)	.1
			Pulmonary artery	5	6.3 (2.2-11.7)	
			Aortic	2	4.5 (3.4-5.6)	

IQR, interquartile range; RV-PA, right ventricle-pulmonary artery.

## Comment

Despite the trend towards performing the repair of the TA at an earlier stage in infancy, the durability of the RV-PA conduits remains as a limiting factor for reaching excellent long-term outcome for this repair. Femoral vein homograft can be used as an alternative, at least in the initial choice of RV-PA conduit, with its comparable durability to pulmonary artery and aortic homograft, as we previously reported.^4^ In this study, we present the comparative mid-term results of RV-PA conduits used for RVOT reconstruction especially in patients with TA. When comparing the 3 conduit groups, although the pulmonary artery homograft had a longer median time to reintervention or replacement of RV-PA conduit, this superiority has not reached statistical significance (*P* = .24). Although a previous study^4^ reported a comparable rate of conduit stenosis and a higher rate of conduit regurgitation for the femoral vein homograft, our study found no statistically significant differences in conduit stenosis, regurgitation, or time to conduit reintervention among the 3 conduit types investigated with limited group size.

Similar to previous studies^2^^,^^5^ linking smaller conduits to rapid degeneration, we observed that conduit size may affect the timing of RV-PA conduit replacement or reintervention, regardless of conduit type. However, these differences were not statistically significant (Table 2).

Larger conduits might be associated with distortion, kinking, and compression of the conduit as well as the branch PAs during sternal closure, leading to regurgitation or stenosis of conduit or branch PAs.^6^ In contrast to the study by Mastropietro and associates,^7^ our outcomes with RV-PA conduit with a z-score exceeding +3 was not associated with early need for conduit or PA reintervention.

However, when a large (> +3 z-score) femoral vein homograft was used, earlier conduit reintervention or replacement was seen compared with aortic or pulmonary artery homografts, although this was not statistically significant in our study. This may be due to the lower elasticity and higher compliance of veins, making femoral vein homografts more prone to dilation, valve insufficiency, and earlier failure.

This study is limited by its retrospective, single-center design, the rarity of the anomaly, and the small sample size. Unequal distribution of conduit types over time and varying follow-up durations across groups also limit comparisons.

In conclusion, optimal-sized femoral vein homografts may serve as a viable alternative to RV-PA conduits in early primary repair of the TA, offering comparable durability to its alternatives. However, further research is warranted to enhance the quality of life for patients with TA by extending the longevity of RV-PA conduits.
