# Supplementary material for: Valved Femoral Vein Homografts as Right Ventricle to Pulmonary Artery Conduit for Repair of Truncus Arteriosus
Source: Ann Thorac Surg Short Rep. 2025 Aug 20;4(1):143–7. doi: 10.1016/j.atssr.2025.07.022 (PMC13100724; doi:10.1016/j.atssr.2025.07.022)
Supplement: Supplementary Material [file mmc1.docx]

SUPPLEMENTARY MATERIAL

**Definitions**

The Modified Collett and Edward Classification was used to define the subtypes of TA. In-hospital (early) mortality was defined as death within 30 days of operation or before hospital discharge. Reintervention was defined as reoperation or transcatheter procedure on the heart or great vessels following TA repair related to the primary repair. While grading RV-PA conduit stenosis/regurgitation, Doppler studies of transthoracic echocardiography were used. Based on the peak Doppler gradient, a gradient of 20-35.9 mm Hg was classified as mild stenosis, 36-64 mm Hg as moderate stenosis, and >64 mm Hg as severe stenosis on RV-PA conduit or pulmonary artery (PA). Furthermore, severe conduit stenosis was also defined as a peak-to-peak gradient above 40 mmHg across the right ventricular outflow tract (RVOT) measured by cardiac catheterization or a peak velocity above 4 m/sec measured by Doppler echocardiogram. Moderate or severe pulmonary stenosis (PS) was defined as significant PS. Transcatheter reintervention or reoperation on RV-PA conduit were performed when symptoms or signs of right heart compromise were present, right ventricle (RV) systolic pressure exceeded 2/3 of the systemic pressure, or there was a severe stenosis or a severe regurgitation of the conduit. Reoperation was considered in cases of conduit or PA stenosis that could not be effectively treated with percutaneous approach. RV-PA conduit regurgitation was graded semi quantitatively as trivial, mild, moderate, or severe by color Doppler echocardiography. Patients who did not present clinical follow-ups within two consecutive years were considered lost to follow-up.

Supplemental Table 1. Demographics and perioperative data

| Variables | | N (%) or median (IQR) |
| --- | --- | --- |
| Female | | 22 (43) |
| Birth weight; kg | | 3 (2.6-3.4) |
| Prematurity | | 8 (16) |
| Antenatal diagnosis | | 26 (51) |
| Known chromosomal abnormality | | 20 (39) |
| DiGeorge syndrome | | 14 (27) |
| Preoperative pulmonary artery banding | | 3 (6) |
| Age at surgery; day | | 7 (5-18) |
| Weight at surgery; kg | | 3 (2.6-3.7) |
| Neonates (<28 days old) | | 40 (78) |
| Below 2.5 kg of body weight at surgery | | 9 (18) |
| Preoperative moderate/severe truncal valve regurgitation | | 9 (18) |
| Associated anomalies | Interrupted aortic arch | 8 (16) |
|  | Hypoplastic arch | 2 (4) |
|  | Major aortopulmonary collateral artery | 1 (2) |
|  | Atrioventricular canal defect | 1 (2) |
| Subtype of truncus arteriosus | Type 1 | 26 (51) |
|  | Type 2 | 22 (43) |
|  | Type 3 | 3 (6) |
| Number of leaflets of truncal valve | Bicuspid | 7 (14) |
|  | Tricuspid | 26 (51) |
|  | Quadricuspid | 16 (31) |
|  | More than four | 2 (4) |
| Coronary artery abnormality | Anomalous origin of left coronary artery | 4 (8) |
|  | Single right coronary artery | 6 (12) |
|  | Intramural left coronary artery | 1 (2) |
| Concomitant procedure | Aortic arch repair | 10 (20) |
|  | Truncal valve repair | 9 (18) |
|  | Atrioventricular canal defect repair | 1 (2) |
|  | Unifocalization | 1 (2) |
| Type of RV-PA conduit | Femoral vein homograft | 29 (57) |
|  | Pulmonary artery homograft | 11 (22) |
|  | Aortic homograft | 7 (14) |
|  | Gore-Tex tube | 3 (6) |
|  | Tube graft with biological valve | 1 (2) |
| Size of RV-PA conduit | Diameter; mm | 11 (9-12) |
|  | Z-score | 2.9 (1.7-3.7) |
| 10 mm or less RV-PA conduit size | | 21 (41) |
| Cardiopulmonary bypass time; min | | 146 (124-177) |
| Cross clamp time; min | | 90 (78-105) |
| Mechanical ventilation time; day | | 6 (5-11) |
| Postoperative need of ECMO support | | 6 (12) |
| Postoperative hospital LOS; day | | 24 (14-41) |

ECMO, extracorporeal membrane oxygenation; kg, kilogram; IQR, interquartile range; LOS, length of stay; min, minute; ml, milliliter; mm, millimeter; RV-PA, right ventricle to pulmonary artery.

Supplemental Table 2. Follow-up data according to the type of RV-PA conduit

| Variables | | | | | | N (%) or median (IQR) | | | |
| --- | --- | --- | --- | --- | --- | --- | --- | --- | --- |
|  |  |  |  |  |  | **Femoral vein homograft n=29** | **Pulmonary artery homograft n=11** | **Aortic homograft n=7** | **P values** |
| Loss to follow-up | | | | | | 5 (17) | 2 (18) | 0 (0) | 0.49 |
| Length of follow-up; year | | | | | | 4 (1-7) | 16 (1-19) | 18 (16-20) | **0.01** |
| RV-PA conduit size at initial surgery | Diameter; mm | | | | | 11 (10-12) | 11 (9-12) | 10 (8.5-12) | 0.6 |
|  | Z-score | | | | | 3 (2.5-3.8) | 2.7 (1.3-3.6) | 2.2 (1.2-4) | 0.19 |
| Catheter-based reintervention on RV-PA conduit before the first RV-PA conduit replacement | Number of patients | | | | | 10 (34) | 2 (18) | 2 (29) | 0.61 |
|  | Time to first catheter reintervention; month | | | | | 9.4 (6-33) | 9.6 (8-11) | 15.5 (9-22) | 0.07 |
| First reoperation on RV-PA conduit | Number of patients | | | | | 15 (52) | 7 (64) | 6 (86) | 0.25 |
|  | Type of implanted conduit | | | Pulmonary | | 10 (34) | 4 (36) | 3 (43) | NA |
|  |  |  |  | Aortic | | 5 (17) | 2 (18) | 3 (43) | NA |
|  |  |  |  | Conduit enlargement | | 0 | 1 (9) | 0 | NA |
|  | Conduit size | Diameter; mm | | | | 20 (18-20) | 19 (18-24) | 18 (17-20) | 0.46 |
|  |  | Z-score | | | | 3 (2-3.8) | 2.3 (2.2-3.1) | 3 (1.8-3.4) | 0.53 |
|  | Time from initial surgery to first reoperation; month | | | | | 27 (18-47) | 71 (32-105) | 27 (21-48) | 0.07 |
| Second reoperation on RV-PA conduit | Number of patients | | | | | 1 (3) | 3 (27) | 4 (57) | **0.002** |
|  | Type of implanted conduit | | Pulmonary | | | 0 | 3 (27) | 4 (57) | NA |
|  |  |  | Aortic | | | 1 (3) | 0 | 0 | NA |
|  | Conduit size | | Diameter; mm | | | 15 (15-15) | 23 (23-24) | 23 (22-24) | 0.29 |
|  |  |  | Z-score | | | 3.3 (3.3-3.3) | 1.2 (0.9-2.1) | 1.6 (1-2) | 0.53 |
|  | Time from initial surgery to second reoperation; month | | | | | 2.8 (2.8-2.8) | 92 (90-104) | 123 (102-154) | 0.15 |
| Third reoperation on RV-PA | Number of patients | | | | | 0 (0) | 0 (0) | 2 (29) | NA |
|  | Type of conduit | | | | Pulmonary | 0 (0) | 0 (0) | 1 (14) | NA |
|  |  |  |  |  | Conduit enlargement | 0 (0) | 0 (0) | 1 (14) | NA |
|  | Conduit size | | Diameter; mm | | | - | - | 25 (25-26) | NA |
|  |  |  | Z-score | | | - | - | 1 (0.6-1.4) | NA |
|  | Time from initial surgery to third reoperation; year | | | | |  |  | 15.7 (15.6-15.8) | NA |
| Catheter reintervention on PA | Number of patients | | | | | 13 (45) | 4 (36) | 5 (71) | 0.34 |
|  | Number of reinterventions | | | | | 21 | 7 | 9 | NA |
|  | Time to first catheter reintervention; month | | | | | 6 (3-12) | 3 (2-4) | 29 (3-46) | 0.23 |
| Right ventricle outflow tract reoperation | | | | | | 0 | 1 (9) | 1 (14) | 0.16 |
| Last follow-up echocardiography of survivals (n=42) | Low left ventricle EF | | | | | 2 (7) | 0 (0) | 1 (14) | 0.48 |
|  | Moderate TV regurgitation | | | | | 1 (3) | 3 (27) | 1 (14) | 0.09 |
|  | Moderate/severe RV-PA conduit stenosis | | | | | 6 (21) | 1 (9) | 2 (29) | 0.57 |
|  | Moderate/severe RV-PA conduit regurgitation | | | | | 6 (21) | 0 (0) | 1 (14) | 0.27 |
|  | RV-PA conduit max gradient | | | | | 31 (18-45) | 29 (28-37) | 26 (25-38) | 0.97 |
|  | RV-PA conduit mean gradient | | | | | 13 (9-18) | 14 (12-18) | 14 (13-23) | 0.79 |
|  | Moderate branch PA stenosis | | | | | 2 (7) | 1 (9) | 0 (0) | 0.74 |
|  | Aortic root z-score | | | | | 3.9 (1.4-5.7) | 4.2 (3.4-5.9) | 3.2 (2.8-3.4) | 0.57 |

EF; ejection fraction, IQR; interquartile, mm; millimeter, PA; pulmonary artery, RV-PA; right ventricle to pulmonary artery, TV; truncal valve. Statistically significant p-values (p<0.05) are displayed in bold.
